# Antigen Reactivity and Clinical Significance of Autoantibodies Directed Against the Pyruvate Dehydrogenase Antigen Complex in Patients With Connective Tissue Disease

**DOI:** 10.3389/fimmu.2022.822996

**Published:** 2022-02-28

**Authors:** Angela Ceribelli, Natasa Isailovic, Carolina Gorlino, Roberto Assandri, Matteo Vecellio, Maria De Santis, Minoru Satoh, Carlo Selmi

**Affiliations:** ^1^Department of Rheumatology and Clinical Immunology, IRCCS Humanitas Research Hospital, Milan, Italy; ^2^Department of Biomedical Sciences, Humanitas University, Milan, Italy; ^3^Clinical Investigation Laboratory, ASST Crema, Crema, Italy; ^4^Department of Clinical Nursing, School of Health Sciences, University of Occupational and Environmental Health, Kitakyushu, Japan

**Keywords:** antimitochondrial antibodies (AMAs), primary biliary cholangitis (PBC), protein immunoprecipitation, IP-Western blot, pyruvate dehydrogenase complex (PDC)

## Abstract

**Introduction:**

Antimitochondrial antibodies (AMAs) are the hallmark of primary biliary cholangitis (PBC) but can be identified also in patients with connective tissue disease, namely, systemic sclerosis (SSc). Protein immunoprecipitation (IP) and IP-Western blot (WB) can be used to confirm AMA positivity directed at the pyruvate dehydrogenase complex (PDC) subunits E1α, E1β, E2/E3, and E3BP in patients showing a cytoplasmic reticular pattern at indirect immunofluorescence when performed in a screening setting before the onset of overt cholestasis in rheumatic patients.

**Patients and Methods:**

We studied sera from 285 patients affected by connective tissue disease [SSc, n = 144; dermato/polymyositis (DM/PM), n = 56; and undifferentiated connective tissue disease (UCTD), n = 85] by indirect immunofluorescence (IIF), protein-IP, and IP-WB to identify specific PDC subunits recognized by AMA.

**Results:**

Twenty percent (57/285) of sera from patients with connective tissue disease had a cytoplasmic reticular pattern at IIF, and in 77% (44/57, including 20 SSc, 12 PM/DM, and 12 UCTD) of these, we detected different titers of autoantibodies against the PDC subunits, specifically against PDC-E2. Among these sera, 4 (9%) tested positive for anti-E1α, 15 (34%) for anti-E1β, and 16 (36%) for anti-E3BP. Four of the 20 AMA-positive SSc cases (20%) had been already diagnosed with PBC, and all were positive for autoantibodies against the subunits PDC-E2, E3, and E3BP.

**Conclusions:**

Using IIF and IP, we confirm that autoantibodies against the PDC components are detected in rheumatic patients with PBC or without liver dysfunction. In view of the strong predictive value of AMA for PBC, a strict follow-up of these latter patients is warranted for an early diagnosis of the disease.

## Introduction

Serum antimitochondrial antibodies (AMA) are directed against several components of the mitochondrial membrane and represent the hallmark of primary biliary cholangitis (PBC) (1). Serum AMA can be detected using routine laboratory techniques, namely, immunoblotting, ELISA, and indirect immunofluorescence (IIF) on rodent kidney/stomach/liver tissue sections (2), usually requested when a clinical suspect of PBC is present. However, AMA positivity has been reported by the International Consensus on Antinuclear Antibody (ANA) Patterns (ICAP; www.autoab.org) as a coarse granular filamentous staining extending throughout the cytoplasm by IIF on cellular substrates such as HEp-2 cells (AC-21), usually performed as a screening test for rheumatic diseases. Thus, AMA presence may be identified by IIF before the clinical suspect of PBC, and this is fundamental for the early diagnosis of PBC and for the correct therapeutic approach before the onset of PBC severe complications such as cirrhosis and liver failure (2, 3). In particular, early AMA identification has been proposed to significantly reduce the need for medical therapies, the severity of PBC clinical features, the need for liver transplantation, and the risk of life-threatening complications (4). Routine commercial tests used for AMA identification, including ELISA and immunoblot, and AMA analysis on triple tissue slides are largely used worldwide for PBC diagnosis but usually when a clinical suspect of PBC is already present (5) while being not routinely used as screening methods for autoantibody (autoAb) analysis as for IIF on HEp-2 slides and immunoprecipitation (IP).

Most PBC-specific AMAs bind to a trypsin-sensitive antigen of the inner mitochondrial membrane previously named M2 (6), mainly targeting the E2 component that forms the core structure of the pyruvate dehydrogenase complex (PDC) in the majority of patients with PBC, but the E1 and E3 subunits are also recognized (7), along with E3BP (initially known as “protein X”) (8). We have previously described the protein-IP pattern of AMA in 15% of our cohort of patients with systemic sclerosis (SSc) (9), showing that AMAs have a strong positive cytoplasmic reticular staining using IIF on HEp-2 cells and not standard IIF on tissue slides, as described also in a recent article by Calise et al. (10).

We report herein the prevalence of serum AMAs in a cohort of patients with different connective tissue diseases, moving from the cytoplasmic reticular pattern detected by IIF on HEp-2 cells (AC-21) and then detecting by IP-Western blot (WB) the different patterns of recognition of AMA of the PDC subunits.

## Materials and Methods

### Subjects

We collected serum samples and clinical information from 285 patients consecutively followed from 2013 to 2019 in our Outpatient clinic for SSc (n = 144) (11), dermato/polymyositis (DM/PM) (n = 56) (12), and undifferentiated connective tissue disease (UCTD; n = 85) (13) according to the most recent classification criteria. None of these patients had been included in our previous study (9). Internationally accepted criteria were used to confirm the diagnosis of PBC (14, 15). Patients who had internal organ involvement consistent with SSc but did not fulfill the American College of Rheumatology (ACR)/European Alliance of Associations for Rheumatology (EULAR) criteria were defined as SSc *sine scleroderma* (16) and very early diagnosis of SSc (VEDOSS) as defined by Minier et al. (17). Pulmonary and cardiac diseases related to the systemic autoimmune disease were defined in our cohort based on established parameters (18, 19). Negative controls are healthy subjects without a history suggestive of systemic autoimmune rheumatic disease and seronegative at our laboratory analysis. As for controls used in the described experiments, in detail, we used (i) cytoplasmic-reticular pattern by IIF (AC-21) and the speckled cytoplasmic pattern by IIF (AC-20) both provided by ICAP as positive controls; (ii) 10 negative controls for each IIF, IP, and IP-WB analysis, as described in the *Materials and Methods* section; (iii) the positive and negative controls for AMA tissue analysis were included in the used kit (Astra Formedic, Milan, Italy).

Liver laboratory tests, i.e., aspartate aminotransferase (AST), alanine aminotransferase (ALT), gamma glutamyltransferase (gammaGT), alkaline phosphatase (ALP), and bilirubin (total and direct), were obtained within 3 months from the blood sampling for autoAbs and, when available, liver histology was also recorded.

The present study was approved by the institutional review board of the Humanitas Research Hospital, and a signed informed consent was obtained from all subjects in accordance with the Declaration of Helsinki and its subsequent modifications.

### Indirect Immunofluorescence

Sera were obtained from whole blood through centrifugation at 2,000g for 15 min and then stored at −20°C until use. Antinuclear and cytoplasmic antibodies were tested by IIF on HEp-2 ANA slides (INOVA Diagnostics, San Diego, CA, USA) using serial dilution of human sera (1:80; 1:160; 1:320; 1:640; 1:1,280) of patients and healthy patients’ sera as control for autofluorescence followed by AlexaFluor488 AffiniPure F(ab′)2 fragment goat anti-human IgG, Fcγ fragment specific (Jackson ImmunoResearch Europe Ltd., Suffolk, UK) as previously described (20). Images were acquired using the Olympus BX53 Upright fluorescence microscope.

After HEp-2 IIF analysis, we performed the IIF analysis on tissue slides for all the patients who had variable expression of autoAbs directed against the PDC, following the manufacturer’s instructions (Astra Formedic, Milan, Italy). The kit included also positive and negative controls, and protocol procedures are the same as described above for IIF on HEp-2 slides. IIF patterns were reported according to the ICAP nomenclature (https://www.anapatterns.org; **Supplementary Table 1**), and we used their reference sera (including AMA) as positive control for autoAb analysis (10).

### Immunoprecipitation-Western Blot

Sera showing a cytoplasmic-reticular pattern by IIF (AC-21) and the control sample that showed speckled cytoplasmic pattern by IIF (AC-20) were tested by IP-WB for AMA component analysis as previously described (9). In detail, 50 μl of candidate sera were cross-linked to the protein-A Sepharose beads using dimethyl pimelimidate (DMP) (Merck KGaA, Burlington, USA) and then immunoprecipitated with cell extract from 5 × 10^6^ K562 cells/sample. Proteins were then fractionated by 8% sodium dodecyl sulfate–polyacrylamide gel electrophoresis (SDS-PAGE) and transferred to a nitrocellulose filter, probed with 1:500 of mouse monoclonal anti-human PDH E1α antibody (Novus Biologicals, Littleton, CO, USA) for a 41-kDa protein identification, followed by horseradish peroxidase (HRP) goat anti-mouse IgG (1:10,000 dilution; Thermo Fisher, Waltham, MA, USA). The same procedure was used to identify the other bands of the complex; in detail, we used mouse anti-human PDH E1β (1:500 dilution; Novus Biological, Littleton, CO, USA) for the 34-kDa protein; mouse anti-human PDH protein X/E3BP (1:1,000 dilution; Novus Biological, Littleton, CO, USA) for the 54-kDa proteins; mouse anti-human PDH E2/E3 proteins of 58 kDa and 74 kDa (1:10,000 dilution; Abcam, Cambridge, UK) followed by goat anti-mouse IgG (Thermo Fisher, Waltham, MA, USA). Signal development was performed by Immobilon Western Chemiluminescent HRP substrate (Millipore, Darmstadt, Germany) and acquired using ChemiDoc (Bio-Rad, CA, USA).

### Protein and RNA Immunoprecipitation and ELISA Validation

Serum autoAbs were screened by protein IP using ^35^S-methionine-labeled K562 cell extract followed by SDS-PAGE and autoradiography and by RNA-IP using unlabeled K562 cell extract followed by urea-PAGE and silver staining, as described previously (20, 21). In the presence of possible autoAb positivity in the controls’ cohort, we used commercial ELISA to validate results from protein or RNA IP to confirm positivity for anti-HMGCoA and -RNA Polymerase III antibodies.

### Statistical Analysis

Continuous variables included in the results were evaluated using Prism version 5.0 (GraphPad Software, Inc., La Jolla, CA, USA). However, statistical comparisons were not included due to the small number of cases in each subcategory.

## Results

**Figure 1:**
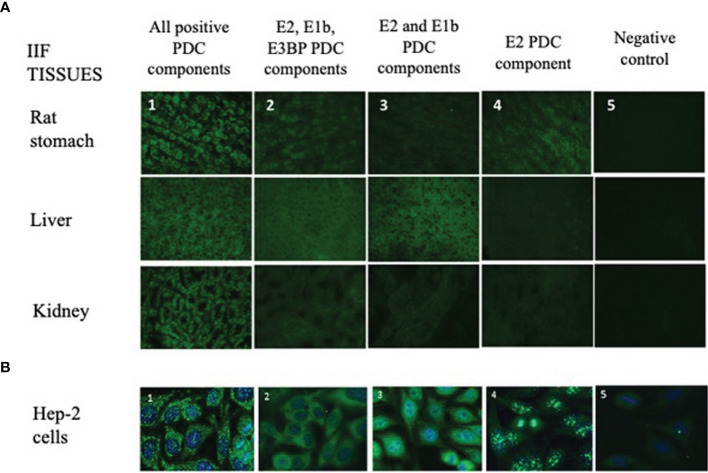
Different IIF patterns of AMA-positive patients on tissue sections and HEp-2 cell slides. **(A)** The IIF analysis on tissues (first row: rat stomach; second row: liver; third row: kidney) is used in routine laboratories as a screening technique of AMA-positive samples, and it shows different IIF intensities concordant with the different expressions of the PDC autoantigens (1, 2, 3, 4, 5; ×40 objective). **1:** IIF pattern of a patient positive for all PDC autoantibodies (E1a, E1b, E2, E3, E3bp). **2:** IIF pattern of a patient with autoantibodies to only E2, E1b, and E3BP components. **3:** IIF pattern of a patient with autoantibodies to only E2 and E1b components. **4:** IIF of a patient positive only for the E2 component. **5:** Negative control. **(B)** Different IIF patterns of AMA-positive sera on HEp-2 cell slides. This method shows different cytoplasmic staining patterns with variable recognition of the PDC antigens (1, 2, 3, 4, 5) using the ×40 objective. These 5 panels correspond to the same 5 panels and patients’ samples shown in panel **(A)**. AMA, antimitochondrial antibodies; IIF, indirect immunofluorescence; PDC, pyruvate dehydrogenase complex.

**Figure 2:**
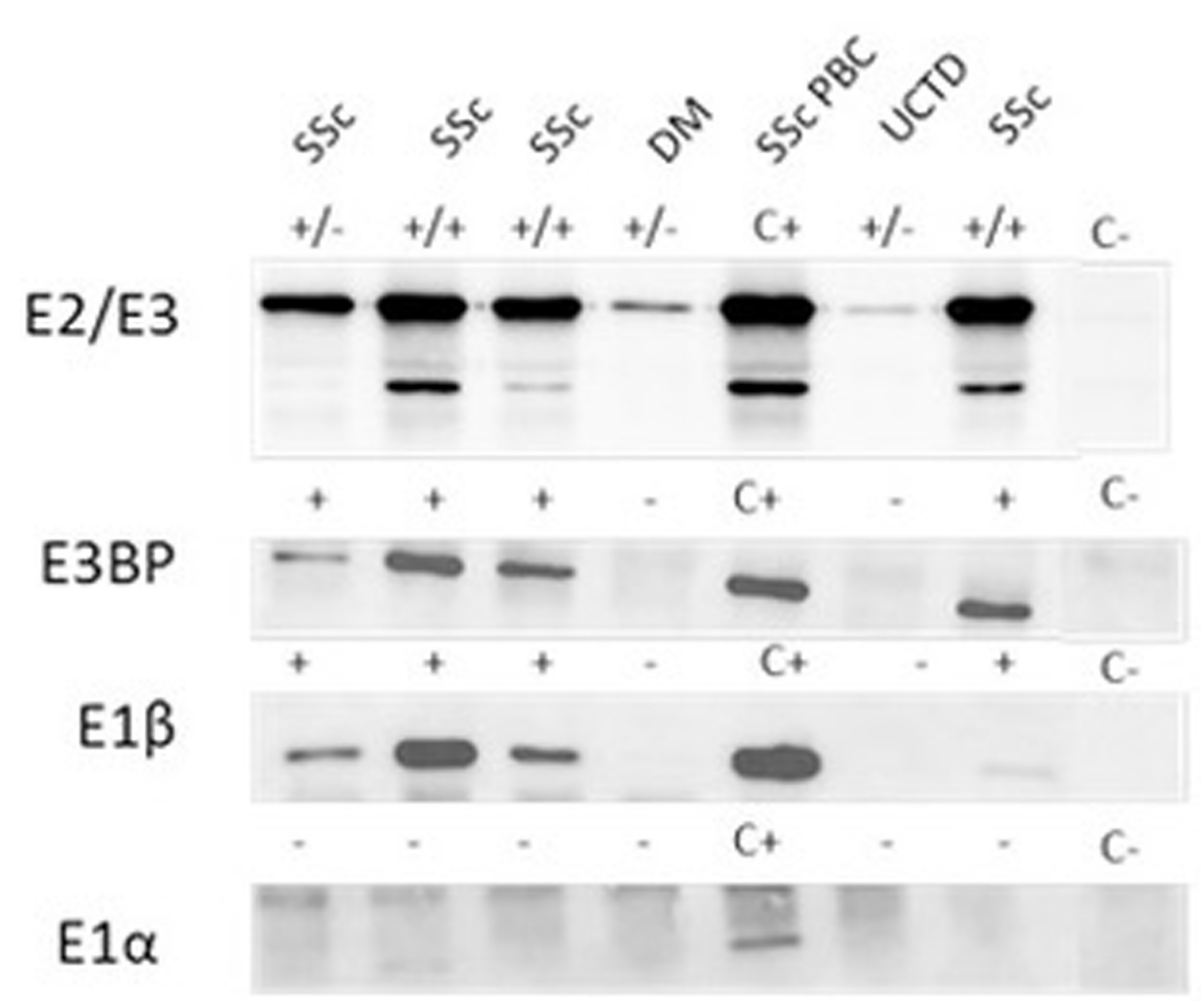
Autoantibody reactivity to PDC antigens by IP-WB. Different reactivities directed against the PDC subunits (E2/E3, E3BP, E1β, E1α) in AMA-positive representative sera from patients with SSc with and without PBC, one patient with DM and one with UCTD. *C+, positive control; C-, negative control; +/- refers to the positivity of only one of the two components of the E2/E3 complex; +/+ both components of the E2/E3 complex are positive; - negative IP-WB band*. AMA, antimitochondrial antibodies; DM, dermatomyositis; IP-WB, immunoprecipitation-Western Blot; PBC, primary biliary cholangitis; PDC, pyruvate dehydrogenase complex; SSc, scleroderma, systemic sclerosis; UCTD, undifferentiated connective tissue disease.

In our cohort, 20% (57/285) of sera had a cytoplasmic reticular pattern at IIF as described both on tissue slides and on HEp-2 cells (**Figure 1**) that we define possibly related to AMA positivity as specified by the ICAP website (AC-21 cytoplasmic pattern, www.anapatterns.org). When analyzing potential differences in IIF patterns between the different IP-WB antibody profiles against the AMA antigens, described in our previous report (9), 44/57 (77%) of these IIF samples showed a variable recognition of the PDC antigens (**Figure 2**), in particular, all sera reacted against the PDC-E2 subunit while a lower variable percentage was directed against the other subunits: 16/44 (36%) against E3BP, 15/44 (34%) against E1β, 8/44 (18%) against E3, and only 4/44 (9%) against the E1α subunit. Moving to the possible clinical significance of these results, in particular, for the known association of AMA and PBC in rheumatic patients affected by SSc, we confirmed the elevated number of SSc patients with these autoAbs, 20/44 (45%) SSc (2 diffuse, 14 limited, and 4 *sine scleroderma*) patients, but we also identified PDC-positive patients with additional diagnosis, in particular, 12/44 (27%) PM/DM (4 DM, 8 PM) and 12/44 (27%) UCTD patients, whose main features are shown in **Table 1**.

**Table 1:** Main feature data of the 44 rheumatic patients with cytoplasmic reticular pattern by HEp-2 IIF analysis, then tested for AMA components.

	SSc (n = 20)	PM/DM (n = 12)	UCTD (n = 12)
**Demographic data**			
Mean age	66.7	61.1	59.8
(years; ± SD)	(± 16.3)	(± 16.4)	(± 16.1)
Women/Men	19/1	10/2	11/1
Mean disease duration	9.8	9.7	9.6
(years; range)	(0–23)	(1–20)	(1–28)
Mean age at disease onset	52.4	53.5	52.1
(years; ± SD)	(± 14.8)	(± 15.1)	(± 14.7)
**Clinical features**			
PBC diagnosis (%)	4 (20)	–	–
Liver laboratory alterations*			
GammaGT	5	1	2
ALT	2	1	2
AST	1	3	3
Total bilirubin	2	–	–
ALP	–	–	–
Cardiac dysfunction	4 (20)	3 (25%)	–
**Laboratory features**			
ANA positivity and additional autoantibodies			
Anti-centromere	14	–	–
Anti-Scl70/topo1	3	–	–
Anti-RNApolIII	3	–	–
Myositis specific antibodies	–	1 Mi2, 1 TIF1gamma, 1 Ku, 1 HMGCoA, 1 ribosomal P	–
Other	–	–	4 Ro/SSA
AMA-positive subunits			
E2 subunit	20	12	12
E1α subunit	3	0	1
E1β subunit	7	2	6
E3 subunit	5	1	2
E3BP/X subunit	8	3	5

*Normal range is evaluated as follows: gammaGT <38 IU/L; ALT <35 IU/L; AST <35 IU/L; total bilirubin 0.2–1.2 mg/dl; ALP 40–150 U/L.

Continuous variables are expressed as mean ± standard deviation (SD) or median (range). AMA, antimitochondrial antibodies; ANA, antinuclear antibodies; DM, dermatomyositis; IIF, indirect immunofluorescence; PBC, primary biliary cholangitis; PM, polymyositis; SD, standard deviation; SSc, scleroderma, systemic sclerosis; UCTD, undifferentiated connective tissue disease.

**Figure 3:**
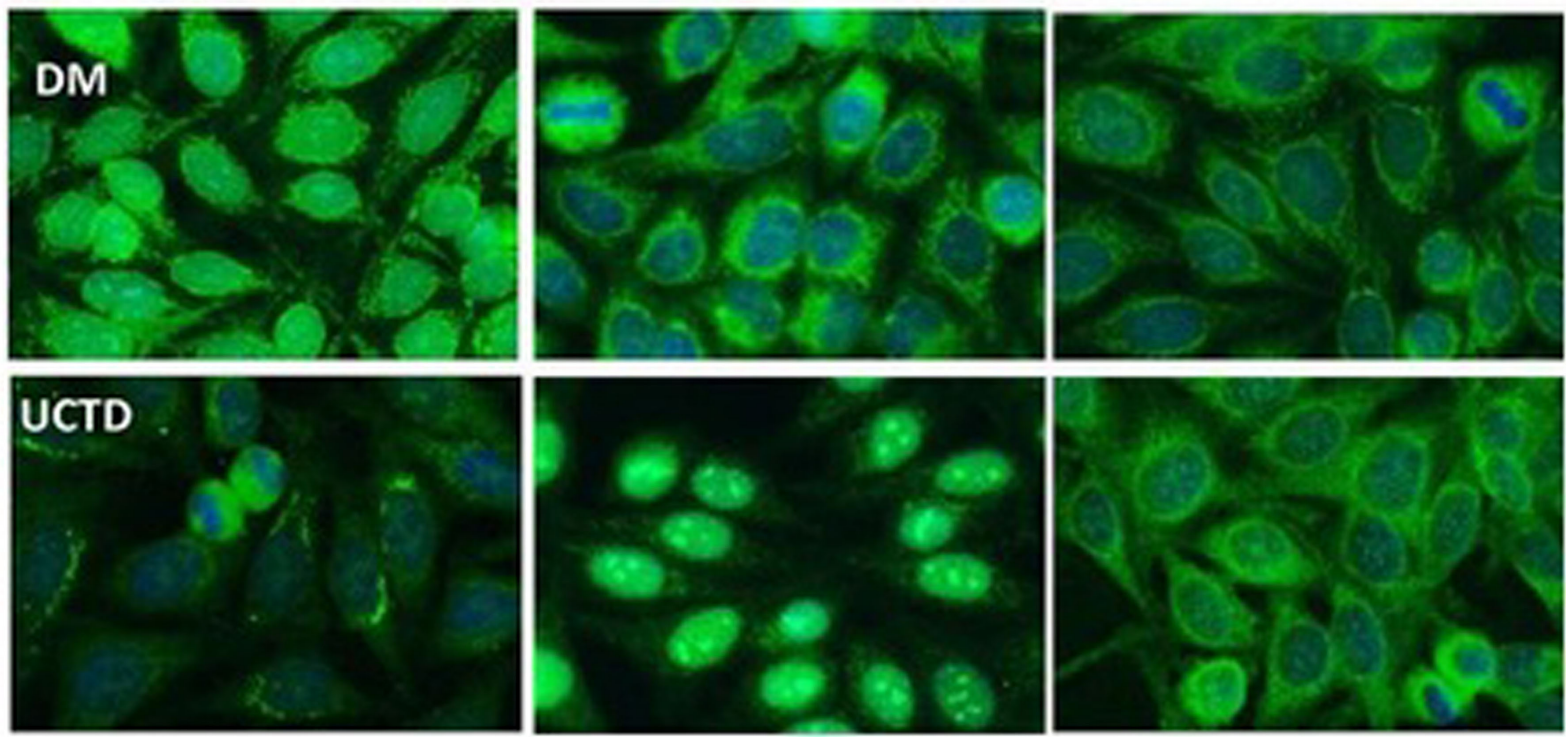
Representative cytoplasmic reticular patterns observed by IIF HEp-2 in AMA-positive patients. In the DM row, we show IIF on HEp2 cells of a DM patient with cytoplasmic staining pattern limited around the nuclei, not spread in the whole cytoplasm as in the case of cytoplasmic reticular AMA-like typical pattern. In the UCTD row, the IIF HEp-2 pattern shows a differential distribution of reticular areas. AMA, antimitochondrial antibodies; IIF, indirect immunofluorescence; DM, dermatomyositis; UCTD, undifferentiated connective tissue disease.

In the SSc cohort with cytoplasmic reticular AMA pattern at IIF (AC-21), 20% (4/20) had a previous diagnosis of PBC undergoing UDCA treatment, while no UCTD or PM/DM case had a confirmed diagnosis of PBC. When performing IIF on tissue slides, we confirmed that all the 4 patients with AMA had the typical IIF pattern on tissue slides. In the group of 44 patients with positive autoAbs against the 5 PDC subunits, 8 (18%) had elevated GGT, 5 (11%) had elevated ALT, and 7 (16%) had elevated AST, while 2 (5%) had increased total bilirubin, and only one patient had increased ALP (**Table 1**) but not enough criteria to fulfill a diagnosis of PBC except for 4 SSc cases who had a defined PBC diagnosis and were in hepatologic follow-up and treatment. In the PM/DM cohort, AMA positivity defined by cytoplasmic reticular IIF (AC-21) and variable expression of PBC components by IP-WB (**Figures 2**, **3****)** were associated with cardiac dysfunction in 1 case each of aortic valve dysfunction, pericardial effusion, and arrhythmia, as previously reported (22).

Further IIF ANA patterns described in AMA-positive subjects are listed in the **Supplementary Table 1**, and they include anti-centromere (ACA) in 14/44 (32%; AC-3), fine nuclear speckled pattern in 8/44 (18%; AC-4), and other patterns (i.e., nuclear large/coarse speckles, nuclear dots, punctate nuclear envelope, clumpy nucleolar, nucleolar homogeneous, polar/Golgi like, and mitotic NuMa-like) in <10% of cases (see **Supplementary Table 1** for ICAP nomenclature). Only two AMA-positive sera (2/44, 5%) were negative for ANA at titers ≥1:160.

Additional autoAbs reported in the 20 SSc patients with variable expression of PDC antigens are anti-Scl70/topoisomerase I antibodies (3/20, 15%), RNApolIII (3/20, 15%), and 1 anti-Th/To positive (1/20, 5%). In the PM/DM and UCTD patients, we detected rare specificities such as anti-Mi2, anti-TIF1γ, anti-ribosomal P, anti-Ku, and anti-HMGCoA in less than 5% of cases each, as confirmed in our research laboratory by combining techniques such as protein IP, RNA IP, IP-WB, and specific ELISA identified according to previous protocols (9, 20, 21).

## Discussion

The first aim of our analysis was to identify AMA-positive samples before the onset of PBC in patients affected by SSc and other rheumatic diseases (1). For this purpose, we started from a screening technique represented by HEp-2 cell IIF to select samples with a cytoplasmic reticular pattern suggestive for AMA as described by ICAP (AC-21 pattern; www.autoab.org). We did not use IIF on tissue slides as screening technique but only to characterize the different expressions of AMA antigens directed against the PDC. Moreover, AMA confirmation was obtained by protein IP as previously described (9). Our results show that a cytoplasmic reticular pattern was present in 20% of rheumatic patients affected in particular by SSc, UCTD, and PM/DM. The identification of AMA by protein IP needs to be confirmed by IP-WB using specific antibodies directed against the different PDC components, as these proteins can have a molecular weight similar to other autoantigens and they need to be further analyzed by IP-WB for confirmation. In fact, PDC proteins have molecular weights around 55 kDa (E3BP and E2), similar to autoantigens such as Ro60, Ro52, or Jo-1 that are common in rheumatic diseases; thus, it is necessary to confirm AMA positivity by IP-WB.

In 2018, the ICAP Committee (www.autoab.org) described a reference serum for cytoplasmic reticular IIF pattern (AC-21 in the ICAP nomenclature) with an antigen association with PDC-E2/M2, BCOADC-E2, OGDC-E2, E1α subunit of PDC, and E3BP/protein X, and we used their standard as positive control for our analysis (10). They described a clinical significance of this pattern as commonly found in PBC, but also in SSc and PBC-SSc overlap syndrome (23), as confirmed in our cohort of rheumatic patients.

In real-life IIF analysis, AMA positivity can be an isolated cytoplasmic pattern with different titers, or it can be associated with additional IIF patterns such as the ANA patterns described in our cohort. Thus, it is necessary to use additional methods to confirm the AMA positivity suspected by IIF through the use of additional tests such as protein IP and IP-WB for each antigenic component recognized by AMA on the PDC. Surprisingly, all our tested patients were positive for autoAbs against E2, the main protein of the PDC on the inner mitochondria membrane (24) that is also the protein used for routine tests worldwide and extended disease-specific immunoassays for the liver profile (6). AMAs also target E1 (composed of the units α and β) and E3 subunits to a lesser extent (25–27) and, similarly, the E3BP component. From a clinical point of view, the majority of PBC patients react with the PDH-E2 component of the PDC targeted by AMA (6), and in our cohort, the second most common autoAb after anti-E2 was anti-E3BP (36%), while E1β follows as third component (34%), and the other components are detected at much lower frequency (8).

As for IIF on tissue sections, this test is routinely used for diagnosis of AMA in clinical laboratories—thanks to its technical simplicity and cost-effectiveness. However, IIF lacks both specificity and sensitivity, and AMA cannot be detected by IIF in up to 10% of PBC patients diagnosed with standard diagnostic criteria (28). These results were confirmed in our analysis, as we observed very low IIF intensity in tissues, especially when not all antigens of the PDC were recognized by the patient’s serum (**Figure 1**). Surprisingly, IIF on HEp-2 slides was more sensitive if taking in consideration the different forms of cytoplasmic reticular patterns to screen for the presence of anti-E2 antibodies, as shown in **Figures 2**, **3**.

AMAs are the hallmark of PBC, and they can be identified in rheumatic patients mainly with diagnosis of SSc (9). In our study, we confirm that half of our tested patients were affected by SSc, and among them, only a small number of cases had liver abnormalities. This aspect is important if we consider that AMA can be identified much earlier than clinical and biochemical manifestations, and in particular, the reactivity with PDC-E2 and/or E3BP seems to be strongly predictive of the presence of PBC (29, 30). On the other hand, no PM/DM patient and UCTD patient have autoimmune liver disease based on routine laboratory testing, and recently, AMA positivity has been reported in association with severe cardiac involvement (such as arrhythmia, myocarditis, and cardiomyopathy) more than PBC in AMA-positive myositis patients (22), even though we observed this association in a limited number of cases in this cohort and in our previous analysis of a larger cohort of PM/DM patients (31).

**Figure 4:**
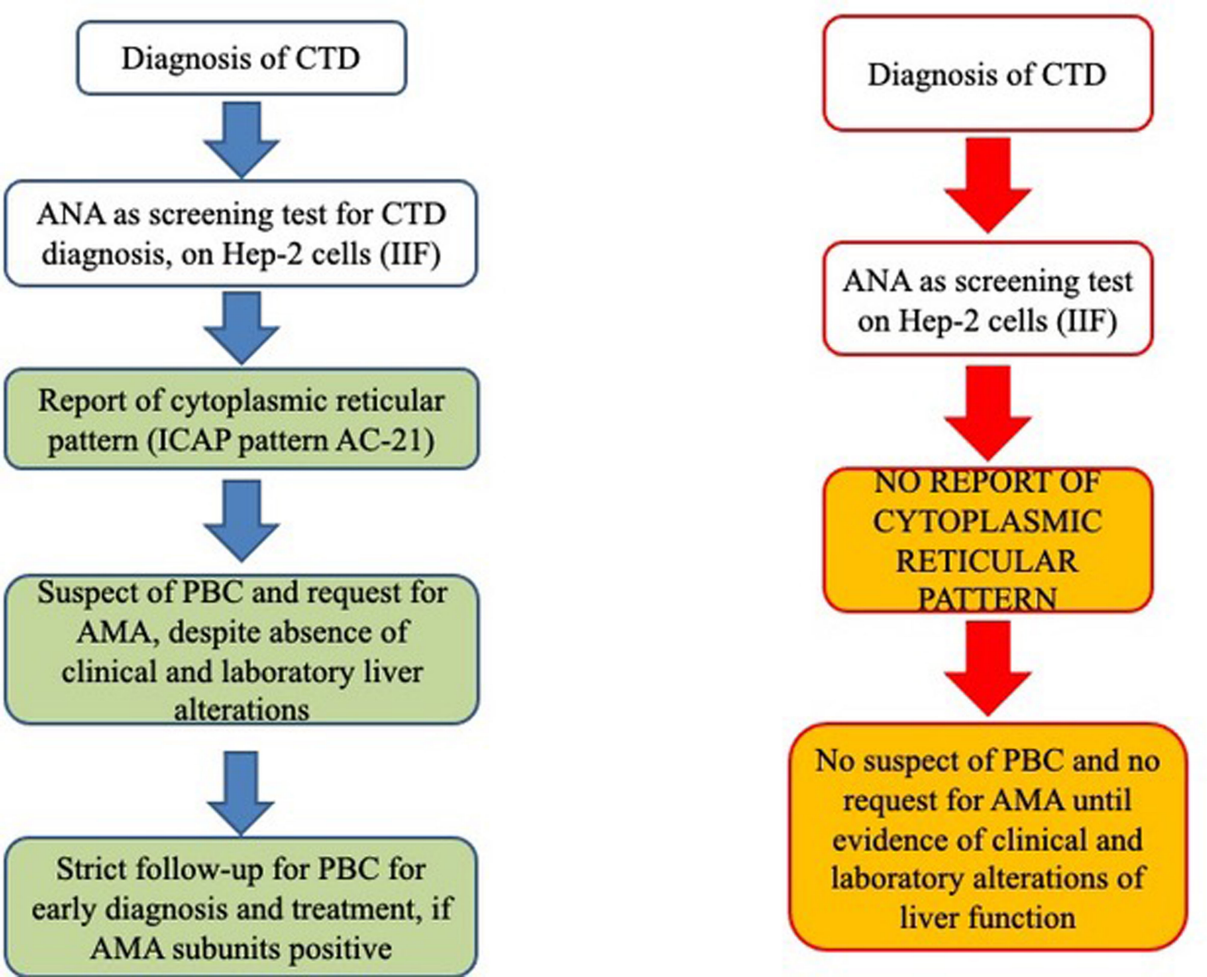
Schematic representation of our proposed working model (left flow chart) from the report of cytoplasmic patterns at immunofluorescence to additional tests for AMA identification, before the onset of clinical features suggestive of altered liver function (right flow chart). AMA, antimitochondrial antibodies; ANA, antinuclear antibodies; CTD, connective tissue disease; ICAP, International Consensus on Antinuclear Antibody Patterns; IIF, indirect immunofluorescence; PBC, primary biliary cholangitis.

In conclusion, as shown in **Figure 4**, we strongly encourage clinical laboratory technicians to report a cytoplasmic reticular staining (AC-21) when analyzing ANA samples of rheumatic patients by HEp-2 cell IIF and to specify any cytoplasmic positivity as described by the ICAP Committee, as this may be the first sign of the presence of AMA. Furthermore, recognition of different components of PDC in AMA-positive PBC patients may define possible clinical association with liver and cardiac disease, and AMA may be detected in rheumatic disease patients without PBC and liver function alterations even years before the clinical manifestations of the liver disease. Thus, AMA positivity needs to be reported to the clinicians even when a cytoplasmic reticular IIF pattern is present (AC-21) to maintain a strict follow-up of these patients for the early diagnosis of PBC or any other liver dysfunction. Then, if IP and IP-WB are available, they may be used for two reasons. First, these methods are used to screen for known and rare autoantigen specificities, from the common ones to the rare autoantigens that may characterize rare diseases such as SSc and PM/DM included in our study, without the need for additional testing. Second, in this screening phase, IP and IP-WB allow the identification of AMAs with the variable patterns described in the present article, thus allowing early diagnosis of PBC (before the clinical suspect based on liver function alterations) on the basis of the different AMA IP patterns. It is clear that when IP and IP-WB cannot be performed, this method must be replaced by conventional methods, but we suggest to ask for these additional tests as soon as possible when the AC-21 pattern is reported in the ANA IIF HEp-2 cell report.

## Data Availability Statement

The raw data supporting the conclusions of this article will be made available by the authors without undue reservation.

## Ethics Statement

The studies involving human participants were reviewed and approved by Humanitas Research Center Ethics Committee, Rozzano, Milan, Italy. The patients/participants provided their written informed consent to participate in this study.

## Author Contributions

Authors equally contributed to the article. All authors contributed to the article and approved the submitted version.

## Conflict of Interest

The authors declare that the research was conducted in the absence of any commercial or financial relationships that could be construed as a potential conflict of interest.

## Publisher’s Note

All claims expressed in this article are solely those of the authors and do not necessarily represent those of their affiliated organizations, or those of the publisher, the editors and the reviewers. Any product that may be evaluated in this article, or claim that may be made by its manufacturer, is not guaranteed or endorsed by the publisher.

## Supplementary Material

Supplementary Table 1 ANA patterns recognized beside the cytoplasmic reticular pattern in 44 rheumatic patients with different diagnosis as described. All the numbers are shown in the total lane are shown as % and absolute number in brackets. *ACA, anti-centromere antibodies; ANA, anti-nuclear antibodies; DM, dermatomyositis; PM, polymyositis, SSc, scleroderma, systemic sclerosis; UCTD, undifferentiated connective tissue disease*.
