# Supplementary material for: Antigen Reactivity and Clinical Significance of Autoantibodies Directed Against the Pyruvate Dehydrogenase Antigen Complex in Patients With Connective Tissue Disease
Source: Front Immunol. 2022 Feb 28;13:822996. doi: 10.3389/fimmu.2022.822996 (PMC8918651; doi:10.3389/fimmu.2022.822996)
Supplement: Supplementary Table 1 — ANA patterns recognized beside the cytoplasmic reticular pattern in 44 rheumatic patients with different diagnosis as described. All the numbers are shown in the total lane are shown as % and absolute number in brackets. ACA, anti-centromere antibodies; ANA, anti-nuclear antibodies; DM, dermatomyositis; PM, polymyositis, SSc, scleroderma, systemic sclerosis; UCTD, undifferentiated connective tissue disease. [file Table_1.pdf]

Supplementary Table 1. ANA patterns recognized beside the cytoplasmic reticular pattern in 44 rheumatic patients with different diagnosis as described. All the numbers are shown in the total lane are shown as % and absolute number in brackets.

**Immunofluorescence patterns based on the ICAP nomenclature (www.anapatterns.org)**	**Total (n=44)**	**SSc**	**PM**	**DM**	**UCTD**
ANA negative	4.6% (2)	0	2	0	0
ACA (AC-3)	31.8% (14)	14	0	0	0
Nuclear speckled (AC-4)	18% (8)	4	1	1	2
Nuclear large/coarse speckled (AC-5)	6.8% (3)	1	0	1	1
Nuclear dots (AC-7)	4.6% (2)	2	0	0	0
Punctate nuclear envelope (AC-12)	4.6% (2)	0	1	1	0
Clumpy nucleolar (AC-9)	4.6% (2)	1	0	0	1
Nucleolar homogenous (AC-8)	4.6% (2)	1	0	0	1
Polar/Golgi like (AC-22)	6.8% (3)	1	0	1	1
Mitotic NuMa-like (AC-26)	2.3% (1)	0	1	0	0

Abbreviations: ACA, anti-centromere antibodies; ANA, anti-nuclear antibodies; DM, dermatomyositis; PM, polymyositis, SSc, scleroderma, systemic sclerosis; UCTD, undifferentiated connective tissue disease
